# From roots to reproduction: the multifaceted roles of rapid alkalinization factor and epidermal patterning factor peptides in plants

**DOI:** 10.1093/jxb/eraf303

**Published:** 2025-07-08

**Authors:** Ran Lu, Judith Lanooij, Elwira Smakowska-Luzan

**Affiliations:** Laboratory of Biochemistry, Wageningen University and Research, Stippeneng 4, Wageningen 6708 WE, The Netherlands; Laboratory of Biochemistry, Wageningen University and Research, Stippeneng 4, Wageningen 6708 WE, The Netherlands; Laboratory of Biochemistry, Wageningen University and Research, Stippeneng 4, Wageningen 6708 WE, The Netherlands; Boyce Thompson Institute, USA

**Keywords:** Arabidopsis, cysteine-rich peptides, development, EPF/EPFL, immunity, peptide, RALF

## Abstract

In plants, peptides play an irreplaceable role as intercellular communication molecules, triggering signal transduction by activating plasma membrane-localized receptors. Of specific interest here are the cysteine-rich peptides (CRPs), which are well-characterized for their disulfide bonds that enhance structural stability and functional specificity. Although the first CRP, systemin, was identified over three decades ago, our understanding of the evolutionary trajectories, functional diversity, and underlying mechanisms of CRPs remains limited. This review focuses on two main families of CRPs: the Rapid Alkalinization Factor (RALF) and the Epidermal Patterning Factor (EPF)/EPF-Like peptides. We thus explore the diverse and, so far, identified signalling pathways in which the peptides have a pivotal function. We organize our tour by providing a comprehensive overview of the discovery of peptides, structural diversity, and biological functions. Particularly, emphasis is placed on their roles in plant growth, development, reproduction, defence against biotic and abiotic stresses, and plant–bacteria symbiosis.

## Teaser

This review explores cysteine-rich peptides, specifically the RALF and EPF/EPFL families, in regulating growth, development, immune responses, and stress adaptation. It highlights recent mechanistic insights and challenges in peptide research.

## Introduction

Plants have to adequately respond to a constantly changing environment by finding the optimal balance between sensing, responding, and coordinating their growth and development (Belkhadir *et al.*, 2014; Brandt and Hothorn, 2016; Couto and Zipfel, 2016). To achieve this, plants have developed mechanisms for coordinating both long- and short-range cell-to-cell communication. These mechanisms include plant-derived hormones and peptides that play key roles in extracellular sensing and signalling (Olsson *et al.*, 2019). These peptides act outside the cell, interacting with specific receptor proteins located at the plasma membrane. This extracellular signalling enables plants to integrate environmental cues with developmental pathways, thereby regulating processes such as growth and development, immune responses, and stress adaptation.

Peptides are typically defined as chains of 2 to 120 amino acids. They are ribosomally synthesized and undergo post-translational modifications, with their biologically active forms often comprising fewer than 20 amino acids (Arnison *et al.*, 2013). Despite significant variations in their amino acid sequences, sizes, three-dimensional (3D) structures, chemistries, and bioactivities, most characterized plant peptides to date are derived from non-functional precursor proteins. These precursor proteins undergo several processing steps before giving rise to the active mature peptide. Depending on their structure, precursor proteins may contain an N-terminal signal sequence for secretion, which needs to be cleaved off before they become mature peptides (pre), an internal pro-domain that must be enzymatically modified to become a mature peptide (pro), or both (prepro) (Tavormina *et al.*, 2015; Olsson *et al.*, 2019). Peptides derived from these non-functional precursors can be categorized into three subgroups based on specific characteristics: (i) peptides undergoing post-translational modifications, such as Pro hydroxylation, Pro glycosylation, and Tyr sulfation; (ii) cysteine-rich peptides (CRPs) containing between 2 and 16 Cys residues; and (iii) non-Cys-rich peptides lacking post-translational modifications but carrying specific amino acids essential for peptide activity, such as Cys, Pro, Tyr, Gly, or Lys (Matsubayashi, 2014; Tavormina *et al.*, 2015). Among these, CRPs stand out for their conserved cysteine residues and functional diversity.

**Fig. 1.**
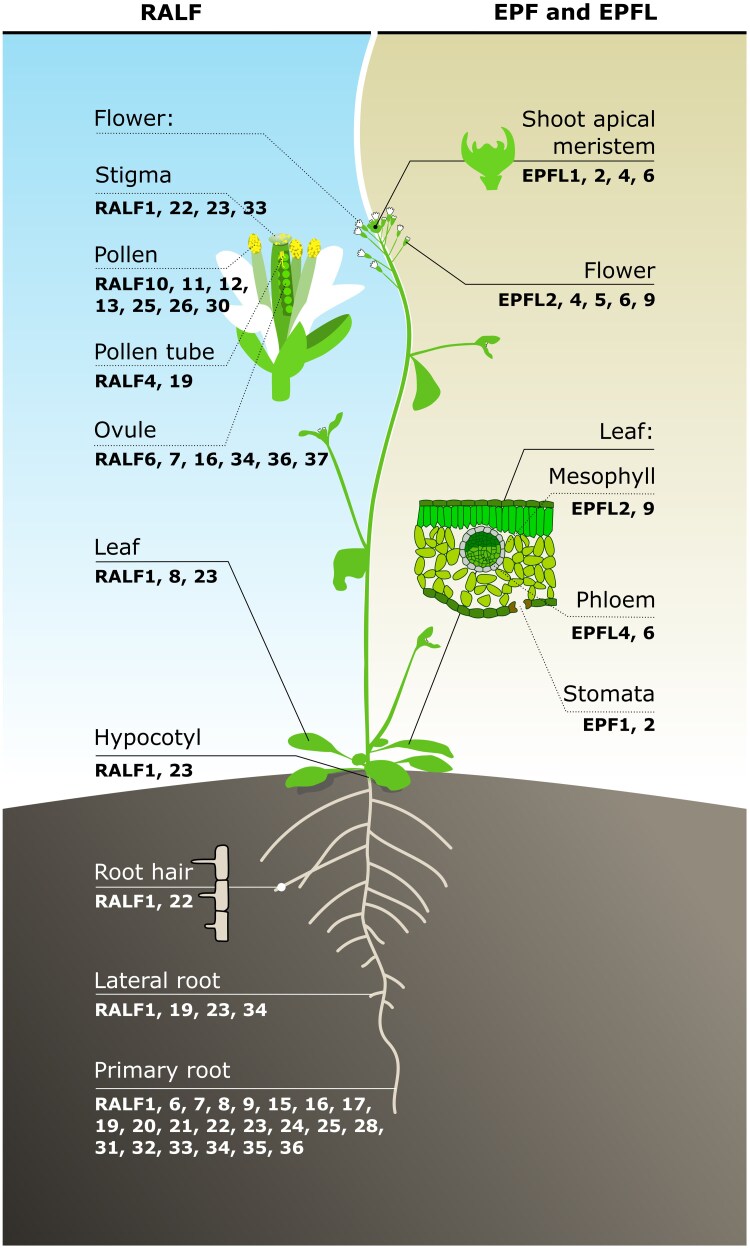
The functional diversity of plant cysteine-rich peptide families, RALF, and EPF/EPFL in Arabidopsis. The representation of various growth and developmental roles of well-characterised functionally RALF and EPF members based on their expression in different Arabidopsis organs. EPF, Epidermal Patterning Factor; EPFL, EPF-Like; RALF, Rapid Alkalinization Factor.

CRPs are a diverse group of small, secreted peptides characterized by conserved cysteine residues, which are predicted to form disulfide bonds and stabilize their 3D structure. These peptides play critical roles during plant development, defence, and symbiosis (Marshall *et al.*, 2011). CRPs are classified into different groups based on sequence and structure similarity and, most importantly, based on the conserved cysteine motifs. Different CRPs have a variety of biological roles. There are, for example, defence-related CRPs, such as defensins (Thomma *et al.*, 2002) and thionins (Bohlmann and Broekaert, 1994), which protect against pathogens. Thomma *et al.*, 2002) and thionins (Bohlmann and Broekaert, 1994), which protect against pathogens. There are also signalling-related CRPs regulating intercellular communication and various developmental processes. Examples of signalling-related CRPs are LURE peptides, which function in plant reproduction (Takeuchi, 2021), RAPID ALKALINIZATION FACTOR (RALF) peptides, which are involved in multiple physiological and developmental processes (Stegmann *et al.*, 2017; L. Liu *et al.*, 2024), and EPIDERMAL PATTERNING FACTOR (EPF)/EPF-Like (EPFL) peptides, involved in regulating multiple developmental processes (Hara *et al.*, 2007; Abrash *et al.*, 2011; Uchida *et al.*, 2011; Kawamoto *et al.*, 2020). Among the diverse CRP families, EPF/EPFL and RALF peptides are the most well-studied CRPs. EPF/EPFL peptides regulate stomatal patterning, inflorescence growth, ovule spacing, and leaf serration through interaction with ERECTA-family receptor (ERf) kinases (Hara *et al.*, 2007; Abrash *et al.*, 2011; Uchida *et al.*, 2011; Kawamoto *et al.*, 2020). In contrast, RALFs influence cell expansion, cell wall remodelling, and immunity through interactions with FERONIA (FER) and related CrRLK/malectin-like receptor kinases (Stegmann *et al.*, 2017; L. Liu *et al.*, 2024) (Fig. 1). Among cysteine-rich peptides, the EPF/EPFL and RALF families have particularly well-characterized signalling pathways. Therefore, they are discussed in detail in this review.

## The diverse roles of RALF peptides in different growth and stress response mechanisms in plants

### Discovery of the RALF peptides

Medium alkalinization of plant cell suspension culture is an early clue of the rapid defence responses triggered in the presence of pathogens or pathogen-derived elicitors (Atkinson *et al.*, 1985; Nürnberger *et al.*, 1994; Boller, 1995; Felix *et al.*, 1999). Strikingly, adding the tomato-derived wound hormone systemin, an 18-amino. Strikingly, adding the tomato-derived wound hormone systemin, an 18-amino-acid polypeptide, at low picomolar levels to tomato (*Lycopersicon peruvianum*) suspension culture cells also induces rapid medium alkalinization (Felix and Boller, 1995; Scheer and Ryan, 1999). Furthermore, another polypeptide that was co-purified with systemin from tobacco (*Nicotiana tabacum*) leaves could also cause rapid extracellular alkalinization (Pearce *et al.*, 2001a, b) That 5 kDa polypeptide, RALF, acts via activating an intracellular mitogen-activated protein kinase. Unlike systemin, however, RALF does not function as a defensive wound-induced signal (Pearce *et al.*, 2001a). The work of Morato do Canto *et al.* (2014) showed that eight out of nine recombinant Arabidopsis RALF (AtRALF) peptides, heterologously expressed in *Escherichia coli*, induced alkalinization in Arabidopsis cells suspension medium. Interestingly, the alkalinization activity is postulated to be coupled with the growth inhibition property exhibited by AtRALF peptides (Abarca *et al.*, 2021). To date, RALFs have been reported to be broadly distributed across more than 50 plant species. Notably, 41 RALF family members have been identified in Arabidopsis, 41 in rice, and 24 in *Lotus* (Campbell and Turner, 2017; Abarca *et al.*, 2021; Jia and Li, 2023; Zhang *et al.*, 2023). Strikingly, RALF peptides are widely expressed in various tissues and organs (Fig. 1), where they play a key role in regulating crosstalk between numerous biological processes, including root development, plant reproduction, plant immune responses and plant adaptation to abiotic stresses (Murphy and De Smet, 2014; Fedoreyeva, 2023).

### Structure and processing of RALF peptides

The precursors of the AtRALF polypeptides consist of 60–140 amino acids and typically include an N-terminal signal peptide that determines the entry into the secretory pathways and a C-terminal mature active RALF peptide (Pearce *et al.*, 2001b; Matos *et al.*, 2008; Srivastava *et al.*, 2009; Matos *et al.*, 2008; Srivastava *et al.*, 2009; Olsson *et al.*, 2019). Based on a single wide-ranging phylogenetic analysis, the RALF family is classified into four major clades (Campbell and Turner, 2017). The clades I, II, and III represent typical RALFs due to key features essential for their activity, including the RR dibasic site, YISY motif and four conserved cysteines. In contrast, clade IV is described as consisting of RALF-related peptides that are highly divergent and lack the conserved motifs of typical RALFs (Abarca *et al.*, 2021; Campbell and Turner, 2017). The conserved motifs play vital roles in the polypeptide maturation and functionality. For instance, a canonical dibasic site (RR) within the predicted subtilase SITE-1 PROTEASE (S1P) cleavage site is essential for the correct proteolytic processing of the peptide precursor in the Golgi apparatus, located just upstream of the presumed start of the mature peptide (Matos *et al.*, 2008; Srivastava *et al.*, 2009; Stegmann *et al.*, 2017). Moreover, a YISY motif is crucial for receptor binding and alkalinization activity (Pearce *et al.*, 2010; Liu *et al.*, 2018; Xiao *et al.*, 2019). In addition, four conserved cysteine residues in the C. In addition, four conserved cysteine residues in the C-terminus are supposed to form two disulfide bridges, which are important for protein conformation and steady activity of RALF (Pearce *et al.*, 2001b; Haruta *et al.*, 2008; Moussu *et al.*, 2020; Abarca *et al.*, 2021).

### RALF involvement in plant root development processes

Peptides regulate multiple aspects of plant root development, including meristem maintenance, the gravitropic response, vascular formation, and lateral root and root hair development (Fedoreyeva, 2023). RALF peptides function as inhibitors of primary root growth across various plant species. Notably, primary root growth inhibitory properties have been demonstrated for 22 out of 34 (approximately 65%) AtRALF peptides (Abarca *et al.*, 2021). The arrest of growth is proposed to be triggered by RALF-induced cell wall alkalization, which results in a reduction in the pH-dependent extensibility of the cell wall and, ultimately, the inhibition of cell expansion (Wolf *et al.*, 2012; Braidwood *et al.*, 2014; Nissen *et al.*, 2016). Among the plant kingdoms, the *Saccharum* spp. Among the plant kingdoms, the *Saccharum* spp. RALF1, *Nicotiana attenuata* RALF (NaRALF), and *Solanum lycopersicum* RALF peptides have also been shown to play crucial roles in inhibiting root growth (Wu *et al.*, 2007; Covey *et al.*, 2010; Mingossi *et al.*, 2010). Studies highlight that RALF22 is a central regulator of root hair cell expansion by serving both as a structural component and as a signalling molecule (Schoenaers *et al.*, 2024). Moreover, the RALF1–FER–EUKARYOTIC TRANSLATION INITATION FACTOR 4E1 (eIF4E) complex mediates polar growth of root hairs by up-regulating the translational levels of RHD6-LIKE 4 (RSL4), while the accumulation of RSL4 provides negative feedback to RALF1, creating a dynamic loop that ultimately suppresses root hair growth (Zhu *et al.*, 2020a, b). Arabidopsis FER is a very well-characterized member of the *Catharanthus roseus* RLK1-LIKE (CrRLK1L) subfamily, which is involved in the processes mentioned above (Chen *et al.*, 2016; Ge *et al.*, 2017; Stegmann *et al.*, 2017; Blackburn *et al.*, 2020). The CrRLK1L receptor family, also known as malectin-like receptor kinases, derives its name from the species in which its first member was identified (Schulze-Muth *et al.*, 1996) and is distinguished by two tandem malectin-like domains in its extracellular domain (Westermann *et al.*, 2019). Interestingly, NaRALF in root hairs and *Physcomitrium patens* (a moss used as a model organism) RALF1/2 (PpRALF1/2) in the rhizoids (root-like structures that anchor moss plants to their substrate) and protonemata (a thread-like, filamentous structure that develops from a germinating moss spore) exhibit similar functions to AtRALF1, promoting tip growth (Wu *et al.*, 2007; Ginanjar *et al.*, 2022). Furthermore, AtRALF1/19/23/34 are implicated in regulating the density of emerging lateral roots (Atkinson *et al.*, 2013; Bergonci *et al.*, 2014; Gonneau *et al.*, 2018).

### Plant reproduction

**Fig. 2.**
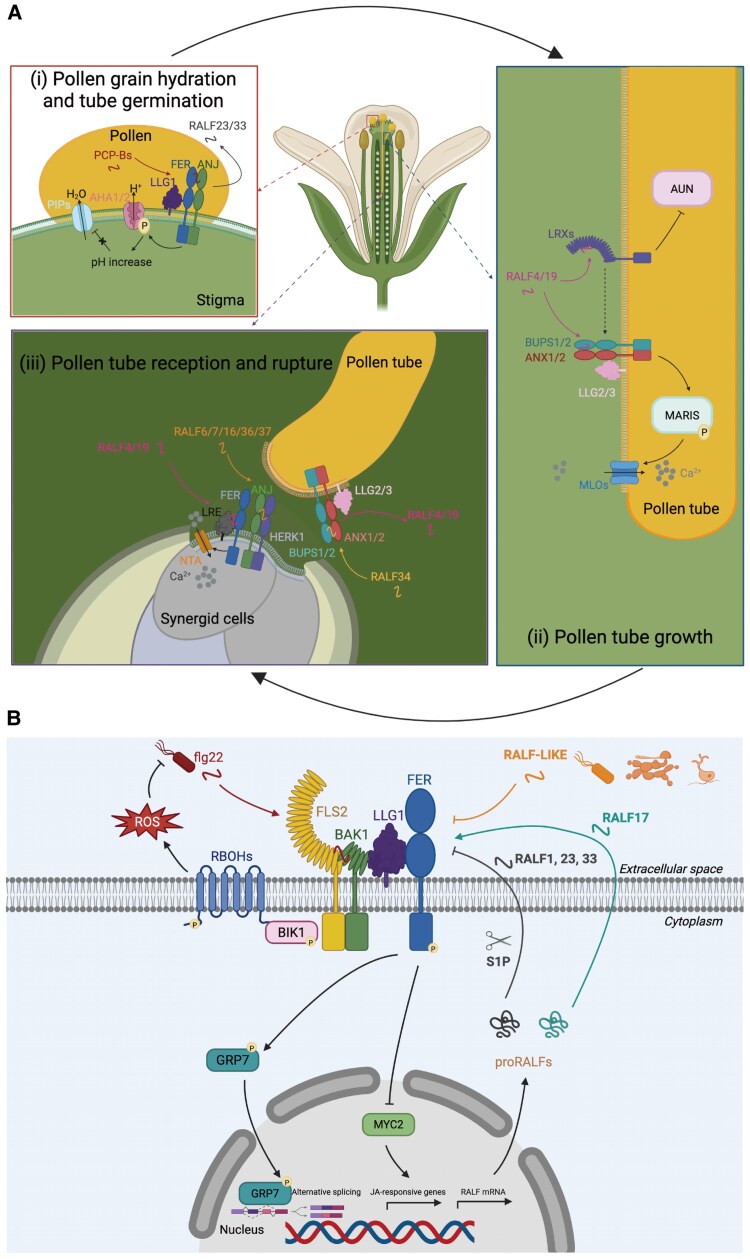
RALF peptides control developmental and defence processes. (A) RALF signalling in different stages of plant reproduction. (i) Upon pollen reception at the stigmatic papilla, PCP-Bs compete with RALF23/33 peptides for binding to the FER–ANJ–LLG receptor complex. This competitive interaction induces phosphorylation of AHA1/2 at activated residues Thr881 and Thr947, leading to H⁺ export into the apoplast. The resulting pH shift alleviates protonation of PIP1/2, triggering aquaporin opening and thereby facilitating pollen hydration and enabling subsequent pollen tube germination. (ii) During the polarized growth of the pollen tube through the style, RALF4/19 peptides activate the cytoplasmic receptor-like kinase MARIS via the ANX1/2−BUPS1/2−LLG2/3 receptor complex, maintaining pollen tube integrity by regulating the Ca²⁺ gradient through activation of the MLO channels in the tip. Simultaneously, the interaction between RALF4/19 and LRX8/9/10/11 is essential for preserving cell wall integrity during pollen tube growth. (iii) Upon reaching the ovule, pollen tube-secreted RALF4/19 peptides are perceived by the FER–LRE receptor module, which recruits the membrane channel protein NTA to induce Ca²⁺ oscillations necessary for pollen tube reception. Ovule-derived RALF34 compete with RALF4/19 for binding to the ANX1/2–BUPS1/2 receptor complex, initiating pollen tube rupture and sperm cell release. Additionally, RALF6/7/16/36/37 contribute to efficient double fertilization by directly interacting with the synergid-located FER–ANJ–HERK1 heteromeric receptor complex. [Created in BioRender; E. Smakowska (2025), https://BioRender.com/uvx0nu7.] (B) RALF signalling in defence responses. In Arabidopsis, the receptor kinase FER and glycosylphosphatidylinositol (GPI)-anchored protein LLG1 controls flg22-induced complex formation between FLS2 and its co-receptor BAK1. The LLG1, a co-receptor of FER, interacts with FLS2 in a flg22-independent manner but binds to BAK1 in a ligand-dependent manner. Upon flg22-triggered activation of FLS2, BIK1 dissociates from the FLS2 complex and directly phosphorylates the NADPH oxidase RBOHD, enhancing ROS generation. Therefore, FER and LLG1 are positive regulators of plant immunity. The FER–LLG1 complex perceives different endogenous RALF peptides, which play antagonistic roles in regulating plant immunity. RALF1, 23, and 33 are negative regulators of immunity in an FER-dependent manner. Their propeptide form is cleaved by S1P, releasing their mature peptide. In contrast, RALF17, which lacks a predicted S1P cleavage site, can induce ROS production and positively regulate immunity dependent on FER. Similarly to endogenous RALF23, pathogen-secreted RALF-like peptides also inhibit plant immune responses in an FER-dependent manner. RALF-dependent inhibition of FER also stabilizes MYC2 through phosphorylation, maintaining the JA synthesis pathway and negatively regulating plant immunity. Furthermore, the RALF1–FER pathway contributes to plant immunity by mediating RNA alternative splicing through FER-mediated phosphorylation of GRP7. [Created in BioRender; E. Smakowska (2025), https://BioRender.com/hue2xg9.] AHA1/2, Arabidopsis plasma membrane H^+^-ATPase1/2; ANJ, ANJEA; ANX, ANXUR; BAK, BRASSINOSTEROID-INSENSITIVE 1-ASSOCIATED KINASE; BIK, BOTRYTIS-INDUCED KINASE; BUPS, Buddha’s Paper Seal; FER, FERONIA; FLS, FLAGELLIN SENSING; GRP7, GLYCINE-RICH RNA BINDING PROTEIN7; HERK, HERCULES RECEPTOR KINASE; JA, jasmonic acid; LLG, LORELEI-like GPI-anchored protein; LRE, LORELEI; LRX, LEUCINE-RICH REPEAT EXTENSIN; MLO, MILDEW RESISTANCE LOCUS O; NTA, NORTIA; PCP-B, POLLEN COAT PROTEIN B; PIP1/2, PLASMA MEMBRANE INTRINSIC PROTEINS 1/2; RALF, RAPID ALKALINIZATION FACTOR; RBOHD, RESPIRATORY BURST OXIDASE HOMOLOGUE D; ROS, reactive oxygen species; S1P, SITE-1 PROTEASE.

In Arabidopsis, RALF peptides are known to guide pollen tube growth within the pistil and to facilitate the pollen tube’s reception by the female gametophyte, ultimately ensuring successful double fertilization (Fig. 2A). A mode of action is proposed for RALF23/33, in which the stigma-secreted peptides inhibit pollen hydration by inducing reactive oxygen species (ROS) production. In case of pollination, however, POLLEN COAT PROTEIN B peptides outcompete RALF23/33 for binding to the FER–ANJEA (ANJ) receptor complex, reducing ROS levels to facilitate pollen hydration and trigger pollen tube germination (C. Liu *et al.*, 2021). Moreover, RALF peptides function as a stigmatic gatekeeper, ensuring that only compatible pollen tube penetration occurs (C. Liu *et al.*, 2021; Lan *et al.*, 2023). In detail, stigmatic RALF1/22/23/33 establish an intergeneric hybridization barrier via CrRLK1L receptor complex FER–CURVY1–ANJ–HERCULES RECEPTOR KINASE 1 (HERK1) and LEUCINE-RICH REPEAT EXTENSIN 3/4/5 (LRX3/4/5), while compatible pollen RALF10/11/12/13/25/26/30 overcome this stigmatic barrier (Lan *et al.*, 2023). During pollen germination and tube growth, RALF4/19 redundantly regulates pollen tube integrity by interacting with the Buddha’s Paper Seal 1/2 (BUPS1/2)–ANXUR1/2 (ANX1/2) receptor complex, with LORELEI-like GPI-anchored proteins 2/3 (LLG2/3) serving as co-receptors (Ge *et al.*, 2017, 2019; Feng *et al.*, 2019). Conversely, RALF4/19 relies on cell wall proteins LRX8/9/10/11 to prevent the accumulation of acidic pectin at the tip of the pollen tube, thereby inhibiting pollen tube growth (Mecchia *et al.*, 2017; Wang *et al.*, 2018). Strikingly, the LRX8–RALF4–pectin interaction exerts a condensing effect, patterning the cell wall’s polymers into a reticulated network that is required for cell wall integrity and expansion during pollen tube growth (Moussu *et al.*, 2023). In addition, RALF4/19 bind to ANX1/2–BUPS1/2–LLG2/3–MARIS to maintain the cytosolic Ca^2+^ gradient in the pollen tube tip and the integrity of the pollen tube through activation of the MILDEW RESISTANCE LOCUS O (MLO) 1/5/9/15 Ca^2+^ channels (Gao *et al.*, 2023). The receptor-like cytoplasmic kinase MARIS operates downstream of CrRLK1L-dependent signalling, acting as a positive regulator of pollen tube integrity and growth (Boisson-Dernier *et al.*, 2015). Throughout the polar elongation of pollen tube tip from style tissue to open spaces, RALF4/19 are essential for BUPS1 to sense increased tensile stress, reinforcing cell wall strength to prevent rupture (Zhou *et al.*, 2021). Moreover, the RALF4/19 peptides, which are recognized by the FER–LORELEI (LRE) module, recruit the calmodulin-gated channel protein NORTIA (NTA) to induce Ca²⁺ oscillations on the synergid cell side, resulting in pollen tube reception (Gao *et al.*, 2022). Upon reaching the ovule, pollen tube-secreted RALF4/19 compete with ovule-secreted RALF34 for binding to the ANX1/2-BUPS1/2 receptor complex, disrupting cell wall integrity and triggering pollen tube rupture to release sperm cells (Ge *et al.*, 2017; Gonneau *et al.*, 2018). Furthermore, another five RALFs (RALF6/7/16/36/37) are reported to trigger and maintain a polytube block at the septum and to regulate pollen tube reception and rupture inside the targeted ovule by physically interacting with FER–ANJ–HERK1 heteromeric receptor complexes (Zhong *et al.*, 2022). In contrast, the functions of RALF members in the reproductive processes of monocotyledonous plants remain largely unexplored. Rare examples are *Oryza sativa* RALF17 (OsRALF17) and OsRALF19, which are homologues of AtRALF4/19, and are essential for pollen tube germination, growth, and integrity (Kim *et al.*, 2023). In maize (*Zea mays*), ZmRALF2/3 regulate cell wall integrity and thickness in growing pollen tubes (Zhou *et al.*, 2024).

### Plant adaptation to abiotic stresses

In Arabidopsis, the RALFs are essential in responding to various environmental stresses, such as high salt and elevated temperature. Overexpressing *RALF22/23* triggers hypersensitivity to salt stress, thus denoting a crucial role of the peptides in salt tolerance. These peptides physically interact with cell wall-localized LRX3/4/5 and plasma membrane-localized FER proteins, forming the LRX3/4/5–RALF22/23–FER complex, which transduces cell wall signals to regulate the salt stress responses (Zhao *et al.*, 2018). Under saline conditions, RALF22/23 dissociates from LRX3/4/5, resulting in internalization of FER, and ultimately in disrupted signal transduction and salt tolerance (Zhao *et al.*, 2018). Functionally, the LRX3/4/5–RALF22/23–FER module regulates salt stress responses by maintaining hormonal homeostasis and controlling ROS accumulation (Zhao *et al.*, 2021). In parallel to RALF22/23, the application of RALF1 induces salt hypersensitivity, in a FER-dependent manner, by inhibiting the activity of plasma membrane H^+^-ATPases, finally leading to Na^+^ and K^+^ accumulation (Yu and Assmann, 2018). Previous work has demonstrated that RALF1 interacts with the FER–glycosylphosphatidylinositol-anchored protein (GPI-AP) LORELEI-like GPI-AP1 (LLG1) complex, where LLG1 functions as a co-receptor for FER and is essential for its cell surface signalling capacity and localization (Li *et al.*, 2015). A recent study on the assembly of pectin–RALF1–FER–LLG1 condensates showed that extracellular RALF–pectin phase separation mediates FER- and LLG1-dependent cell surface responses, triggering a resilience strategy to cope with salt and heat stress (M.C.J. Liu *et al.*, 2024; Rößling *et al.*, 2024). In crops, RALFs also respond to abiotic stresses, allowing the plants to maintain normal growth and yield even in infertile soils. For example, in *Chenopodium quinoa*, RALF15, a paralogue of AtRALF22, is involved in salt stress responses (Jiang *et al.*, 2022). Similarly, knockout lines of *PpRALF3* exhibit reduced sensitivity to both salt and paraquat treatments in *Physcomitrium patens* (Mamaeva *et al.*, 2023). Furthermore, in Tartary buckwheat (*Fagopyrum tataricum*), FtRALF4 regulates responses to low nitrogen conditions (C.Y. Liu *et al.*, 2021). Additionally, in *Phaseolus vulgaris*, PvRALF1 and PvRALF6 jointly regulate the nodulation process and optimize nodule numbers in response to nitrate availability (Solís-Miranda *et al.*, 2023). Finally, in *Oryza sativa*, OsRALF45/46–OsMRLK63–OsRESPIRATORY BURST OXIDASE HOMOLOGUE (OsRBOH) contributes to drought tolerance (Jing *et al.*, 2024).

### Plant immune responses

In Arabidopsis, the bacterial elicitors flg22 and elf18 are derived from flagellin and elongation factor-Tu (EF-TU), respectively (Kunze *et al.*, 2004). They are recognized by FLAGELLIN-SENSING 2 (FLS2) and EF-TU RECEPTOR (EFR) proteins, leading to the formation of a complex with the co-receptor BRASSINOSTEROID INSENSITIVE 1-ASSOCIATED KINASE 1 (BAK1) to trigger ROS bursts and immune-related hormone jasmonic acid (JA) signalling inhibition (Couto and Zipfel, 2016). Notably, ligand-induced complex formation between FLS2/EFR and BAK1 is facilitated by the receptor kinase FER and its co-receptor LLG1 (Stegmann *et al.*, 2017; Xiao *et al.*, 2019). On the one hand, RALF23/33 inhibit the receptor kinase FER function to negatively regulate FLS2/EFR–BAK1 heterodimerization, suppressing flg22/elf18-induced production of ROS and ultimately dampening immune responses (Stegmann *et al.*, 2017). On the other hand, RALF23 induced a LLG1–FER complex to inhibit flg22/elf18-induced ROS accumulation (Xiao *et al.*, 2019). Guo *et al.* (2018) described that FER is involved in the phosphorylation and destabilization of MYC2, a nuclear-localized basic helix–loop–helix leucine zipper transcription factor and a major regulator of JA signalling, thereby inhibiting JA signalling and enhancing the immune response. Strikingly, RALF23-dependent inhibition of FER results in the stabilization of MYC2 and JA accumulation, leading to a decreased defence response to bacteria (Guo *et al.*, 2018). Moreover, lyso-phosphatidylethanolamine may enhance plant immunity against necrotrophs by repressing FER signalling through RALF23 internalization into stress granules, while promoting JA signalling and maintaining ROS homeostasis (Völz *et al.*, 2023). Increasing evidence indicates that RALF–FER signalling plays a pivotal role in plant immunity by recruiting various functional proteins. For instance, the RALF1–FER–Glycine-Rich Protein 7 complex mediates RNA alternative splicing in the nucleus in response to external stimuli (Wang *et al.*, 2020). In parallel to the RALF–FER signalling pathways, RALF22 antagonizes the negative effects on plant immunity by amplifying the Pep3-induced immune response through a significant increase in PROPEP3 transcript and protein levels (Y.H. He *et al.*, 2023). Interestingly, pathogen-secreted RALF homologues enhance infection by promoting pathogenicity and fitness while suppressing host immunity. This process is achieved through interaction with the host plant-encoded FER, which triggers downstream immune-related reactions such as calcium waves, inhibition of ROS bursts, increased mitogen-activated protein kinase (MAPK) phosphorylation, and destabilization of MYC2 (Matsubayashi, 2014; Tavormina *et al.*, 2015). phosphorylation, and destabilization of MYC2 (Matsubayashi, 2014; Tavormina *et al.*, 2015). Overall, the widespread presence of RALF-like sequences across diverse microorganisms highlights the critical role of plant endogenous RALF in modulating immune responses (Masachis *et al.*, 2016; Thynne *et al.*, 2017; Wood *et al.*, 2020) (Fig. 2B).

## The role of EPF peptides in plant growth and development processes

### Discovery of EPF peptides

The first EPF peptide was identified in by Hara *et al.* (2007), through a genome-wide project focusing on identifying peptides regulating plant development in Arabidopsis. Through the overexpression of 153 small open reading frames, they found that gene *AT2G20851* causes decreased stomatal density and named this peptide EPF1 (Hara *et al.*, 2007). Since then, several EPF/EPFL peptides have been shown to be involved in stomatal formation in Arabidopsis (Hara *et al.*, 2009; Hunt and Gray, 2009; Ohki *et al.*, 2011; Lee *et al.*, 2015) and other plant species (barley, Hughes *et al.*, 2017; rice, Caine *et al.*, 2019; bread wheat, Dunn *et al.*, 2019). This knowledge is now implemented by exploring how EPF peptides can be used to improve drought tolerance, through increasing water-use efficiency. This knowledge is now implemented by exploring how EPF peptides can be used to improve drought tolerance, through increasing water-use efficiency.

In Arabidopsis, the EPF/EPFL peptide family consists of 11 members, including EPF1, EPF2 and nine EPFL peptides (Rowe and Bergmann, 2010; Torii, 2012). Phylogenetic analysis groups these peptides into four subfamilies, named after the representative peptides in each clade: EPF1/2/EPFL7 clade, EPFL9/STOMAGEN clade, EPFL1/2/3 clade, and EPFL4/5/6/8 clade. The EPF1/2/7 and EPFL9/STOMAGEN clades are closely related, as are the EPFL1/2/3 and EPFL4/5/6/8 clades (Takata *et al.*, 2013).

### Structure and processing of EPF/EPFL peptides

EPF/EPFL peptides are characterized by the presence of six conserved cysteines at the C-terminal end forming intramolecular disulfide bonds (Kondo *et al.*, 2010; Ohki *et al.*, 2011) and are predicted to contain a secretory signalling sequence at the N-terminal end (Hara *et al.*, 2009). The structure of EPF9/STOMAGEN has been analysed in more detail, and it was shown that different cysteine mutants led to the unfolding and misfolding of the peptide. When two cysteines were mutated to serine, EPFL9/STOMAGEN was correctly folded but was inactive. These observations indicate that the cysteines are critical for maintaining the peptides in stable, active conformation (Kondo *et al.*, 2010). The structure of EPFL9/STOMAGEN and EPF2 was resolved using nuclear magnetic resonance spectroscopy. While the two β-strand regions are conserved across the EPF/EPFL peptide family, the connecting loop exhibits significant sequence variability (Ohki *et al.*, 2011). To investigate if this loop determines functional specificity, it was swapped between EPF2 and EPFL9/STOMAGEN to see if, due to this loop, the peptide is inhibiting stomatal formation (EPF2) or promoting stomatal formation (EPFL9/STOMAGEN). This was indeed the case, as the loop decided whether the peptide promoted or reduced stomatal formation. This suggests that the loop confers functional specificity within the EPF/EPFL peptide family (Ohki *et al.*, 2011).

Before the discovery of the EPF/EPFL peptides and their involvement in stomatal patterning, STOMATAL DENSITY AND DISTRIBUTION (SDD1), a subtilisin-like Ser protease, was suggested to be involved in the proteolytic processing of signal that controls stomatal pattern formation in Arabidopsis. This was based on the observation that the *sdd1-1* mutant had increased stomatal density in leaves (Berger and Altmann, 2000). Therefore, when EPF1 was discovered as a signal controlling stomatal patterning, they checked whether SDD1 acts on EPF1. Based on the observations that the EPF1 overexpression phenotype was independent of SDD1, and that *epf1 sdd1* mutants expressed a more severe phenotype compared with the single mutants (stomatal patterning more disturbed), it was concluded that EPF1 and SDD1 function independently (Hara *et al.*, 2007). To date, no protease has been definitively identified as responsible for the maturation or cleavage of EPF/EPFL peptides in Arabidopsis or other plant species.

### EPF/EPFL peptides, along with ERECTA family receptors, play various roles in plant development

**Fig. 3.**
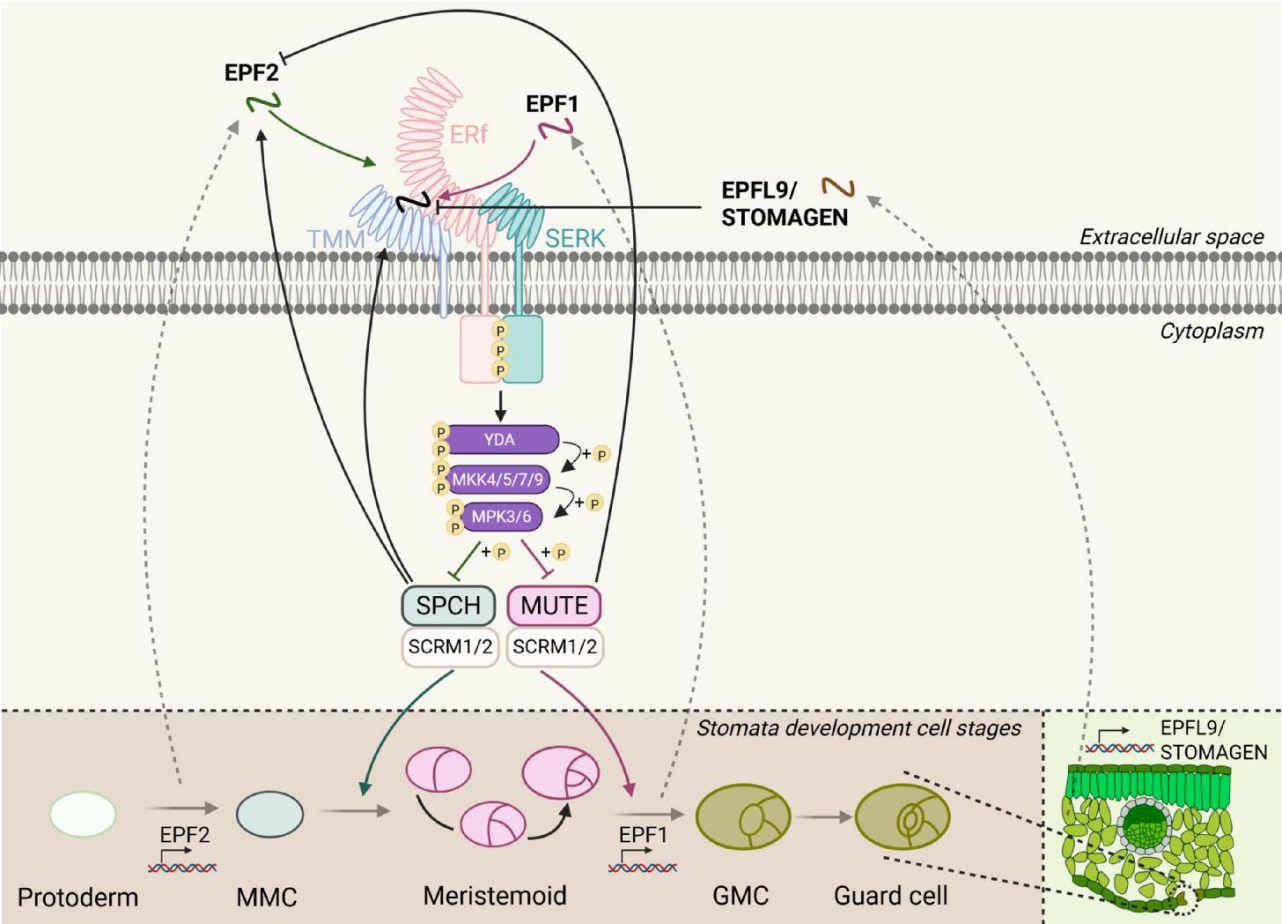
EPF/EPFL peptides control plant development processes. EPF/EPFL signalling in stomata development. EPF1, EPF2, and EPFL9 coordinate stomata development through TMM–ERf–SERK receptor complex, including ER, ERL1, and ERL2, along with their co-receptors TMM and SERKs. EPF1 and EPF2 peptides act as negative regulators of stomatal formation, binding to the TMM–ERf–SERK receptor complex and triggering a MAPK cascade. This cascade involves the MAPKKK YODA (YDA), MAPKK (MKK4/5/7/9), and MAPKs (MPK3/6), which phosphorylate and destabilize the transcription factors SPCH and MUTE, preventing stomata clustering and ensuring proper spacing. EPF2 is expressed in early stomatal precursor cells, such as meristemoid mother cells and meristemoids, regulating stomatal initiation. EPF1 is expressed at later stages in guard mother cells and young guard cells, ensuring stomata are separated by at least one cell for optimal gas exchange. Both EPF1 and EPF2 are part of a negative feedback loop, where SPCH induces the expression of EPF2 and TMM, reinforcing precise stomatal patterning. Conversely, EPFL9/STOMAGEN, a positive regulator of stomatal development, is secreted from mesophyll cells. It binds to the TMM–ERf–SERK receptor complex but does not induce the MAPK cascade, competing with EPF1 and EPF2 and promoting stomatal formation. EPF, EPIDERMAL PATTERNING FACTOR; EPFL, EPF-Like; ER, ERECTA; ERf, ERECTA-family receptor; ERL, ERECTA-LIKE; GMC, guard mother cell; MAPK, mitogen-activated protein kinase; MAPKK/MKK, mitogen-activated protein kinase kinase; MMC, meristemoid mother cell; SCRM, SCREAM; SERK, SOMATIC EMBRYOGENESIS RECEPTOR KINASE; SPCH, SPEECHLESS; TMM, TOO MANY MOUTHS; YDA, YODA. [Created in BioRender; E. Smakowska (2025), https://BioRender.com/li2ki9y.]

The first discovered EPF/EPFL peptides were identified to be important players in stomatal development through regulating a receptor complex composed of ERf, such as ERECTA (ER), ERECTA-LIKE1 (ERL1), and ERECTA-LIKE2 (ERL2), along with their co-receptors TOO MANY MOUTHS (TMM) (Shpak *et al.*, 2005) and SOMATIC EMBRYOGENESIS RECEPTOR KINASES (SERKs) (Cheung and Wu, 2015; Meng *et al.*, 2015). and SOMATIC EMBRYOGENESIS RECEPTOR KINASES (SERKs) (Cheung and Wu, 2015; Meng *et al.*, 2015). EPF1, EPF2, and EPFL9/STOMAGEN peptides control stomatal patterning through the TMM–ERf–SERK receptor complex (Meng *et al.*, 2015). EPF1 and EPF2 are both negative regulators of stomatal development and are secreted from stomatal precursor cells. On the contrary, EPFL9/STOMAGEN is a positive regulator that is secreted from mesophyll cells, thereby promoting stomatal formation when more CO_2_ is needed for photosynthesis (Lee *et al.*, 2015). Although EPF1 and EPF2 are both negative regulators of stomatal formation, they are expressed during different developmental stages. EPF2 is expressed in the meristemoid mother cells (MMCs) and early meristemoids, thereby regulating the initiation of stomatal development (Hunt and Gray, 2009). EPF1 is expressed at a later developmental stage, particularly in guard mother cells (GMCs) and young guard cells, ensuring that stomata are separated by at least one cell for maintaining optimal gas exchange (Nadeau and Sack, 2002). When EPF1 or EPF2 binds to the TMM–ERf–SERK receptor complex, the MAPK cascade is activated. This cascade involves the MAPKK kinase YODA (YDA), MAPK kinases (MKK4/5/7/9), and MAPKs (MPK3/6). These activated MAPKs phosphorylate SPEECHLESS (SPCH) or MUTE. SPCH and MUTE are both transcription factors that promote stomatal formation via heterodimerization with SCREAM1 (SCRM1) and SCRM2, leading to cell fate transition [SPCH: MMC–meristemoid; MUTE: meristemoid–GMC) (Kanaoka *et al.*, 2008). When phosphorylated, SPCH and MUTE are marked for degradation through the 26S proteasome pathway. EPF1 and EPF2 are part of a negative feedback loop, in which SPCH induces the expression of EPF2 and TMM, thereby ensuring proper stomatal patterning and preventing stomatal clustering (Lampard *et al.*, 2008; Horst *et al.*, 2015; Zhang *et al.*, 2015). To the contrary, SPCH is part of a positive feedback loop To the contrary, SPCH is part of a positive feedback loop in which it can bind to its own promoter, thereby maintaining MMC and meristemoid cell fate (Lau *et al.*, 2014; Horst *et al.*, 2015) (Fig. 3). EPFL9/STOMAGEN can bind directly to TMM–ERf–SERK but does not activate MAPK, thereby competing with EPF1 and EPF2 in an antagonistic manner (Lee *et al.*, 2015). Ohki *et al.* (2011) provided evidence that the difference between EPFL9/STOMAGEN and EPF2 being a positive or negative regulator of stomatal development is dependent on the variable loop between the fourth and fifth cysteines of the peptides.

After EPF/EPFL perception by ERf, two U-box E3 ligases, PUB30 and PUB31, are phosphorylated and thereby activated by BAK1, leading to ubiquitination and eventually degradation of ER (Chen *et al.*, 2023). This degradation is tightly regulated by the phosphorylation state of the ER C-terminal tail (ER_CT), which acts as an autoinhibitory domain. Upon ligand binding, transphosphorylation by SERK3/BAK1 modifies ER_CT, shifting it from an inactive to an active state by releasing the receptor kinase inhibitor BRI1 KINASE INHIBITOR 1 (BKI1). This enables signal activation while simultaneously marking ER for turnover. The phosphorylated form of ER_CT preferentially interacts with two U-box E3 ligases, PUB30 and PUB31, thereby promoting rapid receptor degradation following activation. This mechanism prevents prolonged signalling and enables precise modulation of receptor activity, thereby maintaining a balance between effective signal response and regulated receptor turnover (Chen *et al.*, 2025).

EPFL peptides, together with ERf receptors, are also involved in regulating inflorescence growth, using a similar pathway as EPF1 and EPF2 in stomatal development (Abrash *et al.*, 2011; Uchida *et al.*, 2011). EPFL4 and EPFL6 are both secreted from the epidermis and bind to ER, which is expressed in the phloem companion cells. Upon receptor activation in the phloem, the MAPK cascade is activated, which leads to a secondary signal affecting cell proliferation in the surrounding cortex and other stem tissues. This secondary signal is currently unknown. However, it is hypothesized to be a phloem-derived metabolite, such as a polyamine, which are known to influence growth. Alternatively, it could be a phytohormone (e.g. cytokinin or auxin) modulated in the phloem upon ER activation, or a yet-to-be-identified signalling molecule transported from the phloem to cortex cells. Contrary to stomatal development, TMM is not required for EPFL4/6 peptides to activate ER signalling. Instead, TMM inhibits the EPFL4/6–ER signalling, for example in stomatal lineage cells. This inhibition prevents signals from internal tissues (e.g. stems) from unintentionally affecting stomatal lineage cells, ensuring that EPFL4/6-mediated signalling is restricted to regulating inflorescence development (Lin *et al.*, 2017)..

Two EPFL peptides, EPFL2 and EPFL9/STOMAGEN, are involved in ovule spacing and leaf serration. Here, they serve again as ligands for ERf. EPFL9 is expressed in the inner layers of the carpel wall and developing seeds, and apart from functioning antagonistically to EPF2 by preventing SPCH degradation in stomatal development (Lee *et al.*, 2015), it also promotes fruit growth through interaction with ERf (Kawamoto *et al.*, 2020). EPFL9 interacts with the receptor complex but does not activate the MAPK cascade. Instead, it promotes growth by affecting SPINDLY (SPY), a negative regulator of gibberellin signalling. SPY modulates DELLA proteins, which are key repressors of gibberellin-mediated growth (Jacobsen and Olszewski, 1993). This interaction helps facilitate fruit elongation by altering the balance between gibberellin-mediated growth promotion and restriction (Kawamoto *et al.*, 2020). EPFL2 is expressed in the carpel wall and inter-ovule spaces and regulates ovule spacing and density by coordinating auxin during ovule initiation. Through its interaction with ERECTA-like receptors (ERL1 and ERL2) and SERKs, EPFL2 ensures proper auxin distribution, influencing the expression of DORNROSCHEN (DRN), a transcription factor that marks ovule initials. By maintaining regular auxin maxima, EPFL2 establishes precise ovule spacing, minimizing competition, and optimizing seed density (Kawamoto *et al.*, 2020). EPFL2 functions similarly in leaf serration. Here, EPFL2 also modulates auxin responses through ERL1 and ERL2 receptors thereby shaping leaf serration patterns (Takata *et al.*, 2013). The differences in developmental outcomes (ovule spacing vs leaf serration) arise because the effects of EPFL2 are context-dependent, determined by tissue-specific auxin distribution and receptor expression (Tameshige *et al.*, 2016)..

There is quite some redundancy in the EPFL peptide family. Through mutant analysis, EPFL1/2/4/6 have been found to function redundantly in the shoot apical meristem (SAM) and are essential for its proper establishment through ERf (Abrash *et al.*, 2011; Uchida *et al.*, 2011; Kosentka *et al.*, 2019). EPFL1 and EPFL2 are expressed in the boundary region of the SAM and the embryo, and EPFL4 and EPFL6 are expressed in the peripheral zone of the SAM. Based on genetic evidence, EPFL4 and EPFL6 play the primary role in regulating SAM size and leaf initiation, and EPFL1 and EPFL2 contribute. The downstream signalling leading to SAM regulation is currently unknown. This likely involves the MAPK cascade, but the downstream transcription targets have not been discovered yet (Kosentka *et al.*, 2019). Additionally, in another study, these four EPFLs were also found to function redundantly in MMC specification, but it is still unclear how activated ERf leads to restricted MMS formation (Cai *et al.*, 2023). Additionally, EPFL1–6 were shown to play redundant but important roles in regulating integument development together with ERf (Li *et al.*, 2023). EPFL4/5/6 are expressed in filament tissue, act redundantly in stamen development, and are perceived by the ERf–SERK complex. Upon activation of the ERf–SERK receptor complex, the MAPK cascade is triggered, promoting cell proliferation in filaments. The specific transcription factors acting downstream of the MAPK pathway are unknown (Y. He *et al.*, 2023).

EPF/EPFL peptides are conserved across various plant species, playing crucial roles in regulating plant development. In rice (*Oryza sativa*), for instance, the EPF/EPFL peptide family has been identified and characterized, highlighting their significance in plant growth processes (Bessho-Uehara *et al.*, 2016; Xiong *et al.*, 2022). The number of EPF/EPFL genes varies among plant species. In *Oryza sativa*, 13 EPF/EPFL genes have been identified, while *Populus trichocarpa* contains 14 EPF/EPFL genes (S. Liu *et al.*, 2024). This is comparable to the 11 EPF/EPFL genes found in Arabidopsis. To the contrary, cotton species exhibit a higher number of these genes, with 20 in *Gossypium raimondii*, 24 in *G. arboreum*, and 44 in both *G. hirsutum* and *G. barbadense* (Li *et al.*, 2024). Research on the EPF/EPFL peptide family in other plant species is limited, but the conservation of these peptides across diverse plant species suggests their involvement in essential developmental processes. Further investigation is needed to determine if in other plants the EPF/EPF peptide family functions similarly to Arabidopsis.

## Conclusions, challenges, and future directions

In their ever-changing environments, plants constantly battle pathogenic microorganisms while simultaneously attracting beneficial ones. Plants also swiftly sense and respond to various abiotic stresses to ensure their growth and development are not compromised. They have evolved multiple strategies to achieve this, mainly through the sophisticated interactions between peptides and their respective receptors. Plant-derived autocrine peptides, especially cysteine-rich peptides, serve as vital regulatory and signalling molecules for numerous cellular and physiological processes. This review highlights the crucial roles of the RALF and EPF/EPFL peptide families in mediating these processes, emphasizing their contributions to root development, reproduction, stress responses, and immune signalling. Although both families were discovered over two decades ago, it is only in recent years that a deeper mechanistic understanding has emerged regarding how these peptides, along with the sensing receptor, regulate fundamental processes essential for plant survival and proper development.

Numerous challenges and pitfalls hinder research and progress in understanding CRP signalling (De Coninck and De Smet, 2016; Zhong *et al.*, 2019; Ho *et al.*, 2023). For instance, these peptides are known to form disulfide bonds between cysteine residues to maintain their structural integrity. However, these bonds can break under certain unfavourable conditions, such as high temperatures or reducing environments, resulting in peptide degradation and compromised biological activity. Additionally, CRPs may exist as various isoforms or be post-translationally modified, which makes their characterization and functional analysis difficult. Aside from biochemical features, the variable quality of CRPs can also be influenced by differences in synthesis techniques, expression systems, and environmental conditions. Numerous factors, like the presence of other peptides, protein complexes, and the cellular environment, influence the binding affinities and interactions of CRPs with their receptors. Furthermore, producing sufficient quantities of synthetic CRPs can be difficult, especially when complex post-translational modifications are necessary, which are often achievable only in specific expression systems such as those derived from plants, yeast, or bacteria. To address these challenges, a careful experimental design is crucial, encompassing the strategic selection of synthesis, purification, and characterization techniques alongside comprehensive functional assays to assess the roles of CRPs reliably.

RALF peptides play diverse roles in plant development, particularly in root and shoot growth. For instance, they significantly inhibit primary root growth by modifying cell wall mechanics through induced alkalinization, subsequently affecting cell expansion. This regulatory mechanism demonstrates an advanced application of extracellular signalling pathways, where external stimuli and internal developmental processes converge to influence plant cell structure. Furthermore, the impact of RALFs on the development of root hairs and lateral roots showcases considerable adaptability to various growth conditions, enabling plants to flourish across a range of environments. The importance of RALFs also encompasses reproductive processes, particularly in promoting pollen tube growth and fertilization. Acting as ‘stigmatic gatekeepers’, RALFs ensure accurate pollen interactions with female gametophytes, highlighting the essential role of well-regulated signalling mechanisms in successful reproduction. This aspect of RALF function opens exciting avenues for further research into inter- and intra-species reproductive compatibility and hybridization mechanisms. The numerous members of the RALF family are crucial for plant immune responses. In particular, RALF23 influences these processes by regulating the receptor kinase FER. Additionally, RALF homologues released by pathogens can inhibit host immunity, highlighting the intricate interplay between microbial signals and the regulation of plant immunity.

On the other hand, EPF/EPFL peptides play a crucial role in both stomatal patterning and the growth of inflorescences. The dual role of specific EPF peptides, which can act as either negative or positive regulators depending on their functional context, highlights the complexity of stomatal development—an essential process for improving photosynthetic efficiency. Furthermore, the interaction between EPF peptides and ERECTA-family receptors reveals a complex signalling cascade that carefully adjusts stomatal density and distribution in response to developmental signals and environmental factors.

Despite their high degree of structural similarity, these two families of peptides exhibit broad functional diversification. RALFs are more associated with stress responses and cell growth modulation, whereas EPFs/EPFLs are central to epidermal patterning and stomatal development. RALFs primarily alter ion dynamics and pH, while EPFs/EPFLs focus on regulating cell fate decisions and patterning through kinase-mediated pathways.

There is limited knowledge about the evolution of peptide families. The work of Takata *et al.* (2013) demonstrates that EPF/EPFL genes are conserved across land plants but are absent in algal species, indicating that their emergence coincided with the transition of plants to terrestrial environments. The EPF/EPFL family expanded via tandem and segmental duplications, especially in angiosperms. It is also indicated that the emergence of EPFL9/Stomagen-like genes in vascular plants may have contributed to the increased stomatal density observed during the Devonian period, facilitating improved gas exchange and supporting the evolution of larger leaf structures. As with the EPF/EPFL peptides, RALF peptides are also present across various land plants but are absent in green algae, so their emergence also probably coincided with the transition of plants to terrestrial environments. The RALFs exhibit widespread duplication across land plants, with a notable expansion in species such as Arabidopsis, which possesses 37 RALF genes (Campbell and Turner, 2017).

Despite substantial advancements in understanding the roles of RALF and EPF peptides, numerous questions remain. The specifics of downstream signalling pathways, co-receptor involvement, and the interactions between various peptide signalling systems need further exploration. Additionally, the evolutionary conservation of these peptide families across diverse plant species suggests that their functions may extend beyond what has been defined in model organisms such as Arabidopsis.

## Data Availability

No data available, review article.
